# Conceptualisation and Role of Market Access in Pharmaceutical Industry: A Scoping Review

**DOI:** 10.3390/jmahp12020007

**Published:** 2024-05-01

**Authors:** Clara Fatoye, Gillian Yeowell, Eula Miller, Isaac Odeyemi, Chidozie Mbada

**Affiliations:** 1 Department of Health and Social Care, University Campus Oldham, Oldham OL1 1BB, UK; clara.fatoye@oldham.ac.uk; 2 Department of Health Professions, Manchester Metropolitan University, Manchester M15 6BH, UK; g.yeowell@mmu.ac.uk (G.Y.); iodeyemi@aol.com (I.O.); 3 School of Nursing and Public Health, Manchester Metropolitan University, Manchester M15 6BH, UK; e.miller@mmu.ac.uk

**Keywords:** market access, pharmaceutical industry, professional, scoping review

## Abstract

Background: Understanding the concept and dynamic process of the evolution of professional identity and roles of market access (MA) in the pharmaceutical industry (pharma) is critical to personal, interpersonal, and professional levels of development and impact. Objective: The aim was to carry out a scoping review of the conceptualisation of MA within pharma. Data Sources: BioMed Central, WorldCat.org, and Directory of Open Access Journals were searched from 2003 to 2023. Study Selection: All articles on concepts or definitions and other surrogate terms on MA in pharma were selected. Data Extraction: Keywords generated from an initial cursory literature search on MA in pharma were used in conjunction with AND/OR as search terms. Using the data charting method, key findings were mapped and summarised descriptively. inductive analysis was performed, allowing codes/themes that are relevant to the concept to emerge. Data Synthesis: Arskey and O’Malley’s six-stage framework and the PRISMA extension for scoping reviews extension checklist were used as the review and reporting templates. The databases search yielded 222 results. Following title and abstract screening, a total of 146 papers were screened, and 127 of them were excluded. Full-text review was conducted for 19 papers that were deemed by two reviewers to meet the eligibility criteria. One of the authors arbitrated on disputed papers for inclusion. Only 14 of the included papers were found to meet the criteria for the final analysis. Five conceptual dimensions of MA in pharma were identified as “right products”, “right patient”, “right price”, “right point” (time), and “right place” (setting). Conclusions: Market access in pharma is a process that commences with the development and availability of the right products that are proven to be efficacious and disease/condition-specific (including medications, medical devices, and vaccines); specifically produced for the right patients or end users who will maximise best clinical outcomes and economic value; delivered at the right point in a timely, sustained, and efficient manner, given at the right price (commercially viable or reimbursed price that represents good value); and conducted within the economic, policy, societal, and technological contexts, with the overarching goal of achieving the best patient outcomes and ensuring product profitability.

## 1. Introduction

From a broad perspective, the phrase “market access” (MA) was first used by the World Trade Organisation (WTO) to characterise the competitive interaction between a nation’s domestic and imported commodities [1]. The WTO conceptualise MA as representing unlimited access to the whole market in any given country, where one can sell a product and make money [1]. Further, the WTO defines MA according to goods (commodities) as the “conditions, tariff and non-tariff measures, agreed by members for the entry of specific goods into their markets” [2]. Subsequently, MA has been considered differently to reflect the peculiar characteristics of the contexts within which it is being applied. For example, in international trade, MA is a company’s ability to enter a foreign market by selling its goods and services in another country [3]. However, MA within the healthcare sector is primarily related to the pharmaceutical sector, making it distinct from normal goods as reflected by the WTO, which is primarily regulated through the interaction of supply and demand for goods and services [4]. Even though there are many similarities between healthcare items and other goods in a free market economy, the healthcare market poses a challenge to the traditional economic paradigm, as the interplay of demand and supply is not the same [4].

The challenges facing MA in pharma include obtaining market access authorisation (MAu), pricing and reimbursement (P&R) levels, logistics (storage and supply circumstances), drug surveillance (following-up on potential and actual product adverse effects), and ensuring access to pharmaceutical products for the patients [4,5]. While the process of obtaining MAu from a regulatory agency to make the product accessible to all indicated patients is based on consideration of the product’s safety, efficacy, and quality obtained from findings of randomised clinical trials [4], the pricing regulatory process for pharmaceutical products are country-specific, as there is no standardised way of doing this [4]. Nonetheless, the pharmaceutical business seems to have mastered all these challenges except P&R in actual practice [6]. In the pharmaceutical industry, it has become increasingly necessary to satisfy the value perceptions of various stakeholders, particularly payers, to gain MA for goods as opposed to the traditional requirement of just persuading regulators of a product’s safety and efficacy. Hence, there is a need to understand the peculiarity of MA within pharma.

Within pharma, MA is a lay term for efforts that ensure patients have access to pharmaceutical products (including medications, medical devices, and vaccines). Thus, it involves making pharmaceutical items available to patients who are end users [4]. The grey literature reveals several attempts at defining MA in pharma. Some of these definitions include the following: “MA is about getting the right treatment to the right patient at the right time, and possibly even at the right price” [7]; and “… MA refers to a company’s ability to provide appropriate treatments to patients—consistently, continually, and quickly” [7,8]; and “MA, put simply, is the process to enable patients to receive appropriate treatment at the right time and at a price that represents good value” [5,9].

Within the research literature, Sendyona and colleagues [10,11,12] defined MA as the process that guarantees the development and commercial availability of pharmaceutical products with appropriate value propositions, resulting in their prescription and successful uptake decisions by payers and patients with the ultimate goal of achieving profitability and the best patient outcomes. Still, these authors concluded that “the concept of MA is still poorly understood, and the definition varies depending on the stakeholders’ perspectives” [12]. It is adducible that this lack of understanding of how MA is conceptualised has resulted in a lack of consistency in roles or activities within and across different pharmaceutical industries and across countries/regions, which may have hindered the availability of a ubiquitous or standard definition of MA to different contexts within pharma. A shared understanding of how MA is conceptualised and defined is needed to ensure accessibility to pharmaceutical products for patient benefit.

## 2. Materials and Methods

A scoping review was used in this study to map themes on the conceptualisation of MA and its role within pharma. This scoping review was registered on the Open Science Framework (OSF) registries: https://osf.io/qs7b6 (accessed on 12 March 2024).

A scoping review is a relevant approach to exploring a broad variety of literature from many sources on an emerging subject matter. As the body of literature on MA in pharma seems to exhibit a complex and heterogeneous nature not amenable to a systematic review, this approach was deemed the most suitable to answer the research question [13].

This scoping review was conducted in line with Arskey and O’Malley’s [14] six-stage framework for conducting scoping reviews and also based on the recommendations by Levac et al. [15] and the Joanna Briggs Institute (JBI) manual for evidence synthesis [16]. The Preferred Reporting Items for Systematic reviews and Meta-Analyses (PRISMA) extension for scoping reviews extension checklist was adopted to guide the reporting of this scoping review [17].

The six stages of the Arksey and O’Malley [14] framework indicate the activities and steps that should be followed. These involve to (i) specify the research question, (ii) identify the relevant literature, (iii) select studies, (iv) map out the data, (v) summarize, synthesize, and report the results, and (vi) include expert consultation.

***Stage 1: Identifying the research question.***

The research question was how is MA conceptualised and its role defined within pharma?

***Stage 2: Identifying the relevant literature.***

*Search strategy for databases*

The search strategy was developed based on a cursory literature search and critical discussion with the research team and university librarian as well as in consultation with pharma experts. The search strategy was piloted and refined as appropriate. The following databases were used for the search—BioMed Central, WorldCat.org, and Directory of Open Access Journals. Keywords were generated from a cursory literature search on MA in pharma and were used in conjunction with AND/OR as search terms for piloting. The search was undertaken from January to February 2023. All databases were searched from inception.

*Pilot search of the databases*

The search terms used for pre-pilot testing in conjunction with AND/OR, were market access, MA, payer market access, market access strategy, pharmaceutical, Pharms*, drug, definition, character*, descri*, concept* perce*, and meaning. Following the pretesting, further consultation was undertaken with experts in industry and library staff in order to refine the search strategy. Filters were used in the search to include peer-reviewed (full manuscripts and conference papers) articles published in the last 20 years (2003–2023) and for all papers written in the English language only. Applying the filters helped to reduce the result yields (from over 1 million). Additionally, the search year of 2003 was intentionally chosen to coincide with the time when MA negotiations were first introduced [18]. Articles on MA in which full scripts were not accessible were excluded.

***Stage 3: Study selection.***

One researcher (C.F.) independently assessed titles and abstracts of the studies retrieved using the outlined search strategy. A second researcher (G.Y.) repeated the process on 100% of the records retrieved to verify the search. A third reviewer (C.M.) was available to arbitrate and make the final decision should there be any disagreements (Arksey and O’Malley [14]. Full-text records that met the eligibility criteria were included in the review.

***Stage 4: Charting the data.***

Data extraction (referred to as “charting the data”) was performed for all the included articles to create a descriptive summary and thematic presentation of the findings that addresses the study’s research question. Charting was undertaken as a crucial component of the scoping review process using a charting form developed by the researchers. The charting allowed for the condensing of vast volumes of data into a format that was simple to comprehend, enhanced data visualisation, and enabled meticulous notes of each identified study to be made and references tracked. The chart form used in this review was used to extract information about the authors, setting or country of study, how MA was conceptualised, how MA was defined, type of pharmaceutical product, MA strategy, and disease area.

***Stage 5: Collating, summarising, and reporting the results.***

Using the data charting method, key findings were mapped and summarised. All eligible articles on MA in pharma were read, and core attributes of features related to the concepts or definitions (i.e., MA) and other surrogate terms were extracted. To achieve familiarisation or immersion in the raw data and to be able to derive the key attributes of conceptualisation and definitions of MA, one researcher (C.F.) reviewed the data several times. Overall, the data were descriptively summarized, and inductive analysis was carried out by reading through the data and allowing codes/themes that were relevant to the concept to be identified. As assessment of the methodological quality of the literature is not required for scoping reviews [13,15], to ensure objectivity and trustworthiness as well as reduce bias, the analysis process was reviewed by the other researchers (C.M. and G.Y.).

***Stage 6: Consultation exercise.***

One of the researchers (C.F.) consulted experts (*n* = 2) within pharma and an academic librarian on key terms and themes relevant to the literature search and analysis of the findings.

## 3. Results

**Figure 1:**
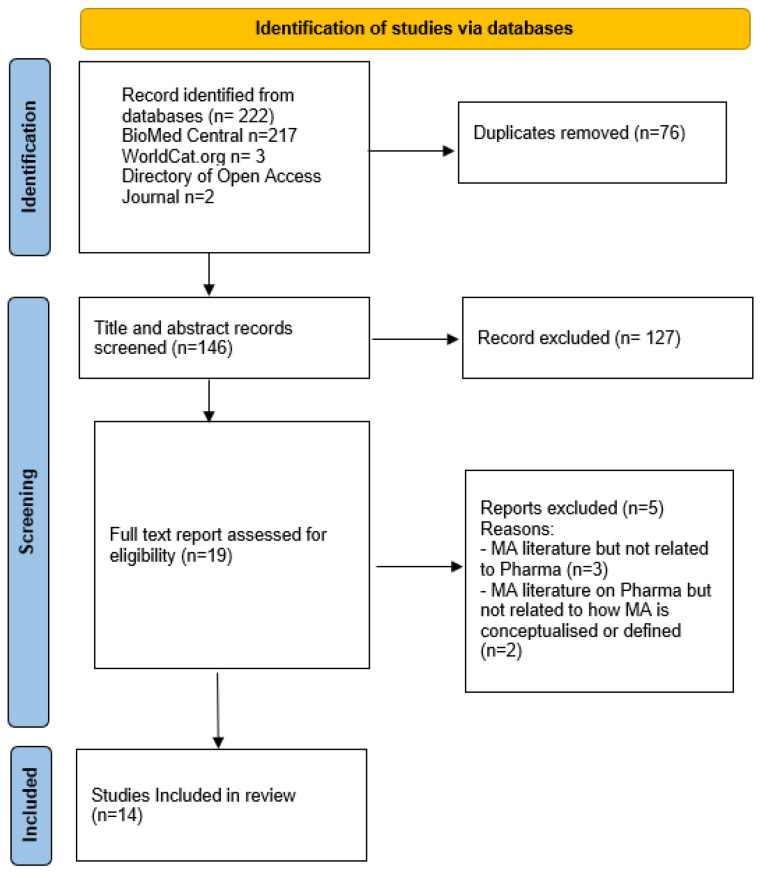
PRISMA flow diagram for scoping review.

The PRISMA extension for scoping reviews (PRISMA-ScR) diagram was used to present the conduct of this review (Figure 1). The result of the databases search yielded 222 hits (BioMed Central n = 217, WorldCat.org n = 3, and Directory of Open Access Journal n = 2. Following title and abstract screening, a total of 146 papers were screened, and 127 of them were excluded (Figure 1). Full-text review was conducted for 19 papers that met the eligibility criteria. There was 95% concordance between the two reviewers (C.F. and G.Y.). The third reviewer (C.M.) arbitrated on the one study where there was disagreement. From this process, five papers were excluded, leaving a total of 14 papers for inclusion in the final analysis.

Table 1 presents the characteristics and summarises the included studies in this scoping review in terms of settings, countries, type of pharmaceutical products (PP), and how MA was conceptualised and defined. The studies included were from different geographical regions. Three of the studies were from Africa [19,20,21]; four were from Europe [22,23,24,25]; three from North America [26,27,28]; two were from Asia [29,30], and two studies stated that their research covered low-income countries [31,32], with Vialle-Valentin et al. [32] using Rwanda as its case study for low-income countries. The earliest paper included in the review was published in 2008 [32], and the most recent paper was published in 2021 [19]. Three studies had global reach and were not limited to a particular geographical region [22,24,31] (Table 2). Some of the studies were carried out in community settings [19,20,21,25,27,29], while others were in the hospital settings [23,26,28,30]. One of the studies had no specific study setting [32]. All the studies focused on drugs as a pharmaceutical product, except for the study by Romao et al. [25], which was on a medical device (Table 1).

Twelve out of the fourteen studies included in this review conceptualised patients having access to PP through various MA strategies. Schmittdiel et al. [27] conceptualised MA in terms of the cost of drugs and how it can affect access and medication and adherence. Simon et al. [28] conceptualised MA as computerised provider entry (CPOE) to ensure safety, quality, and efficiency for patients (Table 1). Three studies explored how patients can have better access to malaria drugs/treatment [20] (Tanzania); Patouillard et al. [29] (Cambodia); and Rutta et al. [21] (Tanzania)). Lee et al. [31] and Miller et al. [26] both explored MA in terms of access to PP for patients living with HIV, while Schmittdiel et al. [27] explored access among patients with diabetes in receipt of Medicare. Their study aimed to examine communication between patients and their physician by focusing on how the cost of medication can affect the uptake of PP. Romao et al.’s [25] study aimed to evaluate access to ostomy products and ostomy patients’ and caregivers’ satisfaction with their pharmacies. Ameh et al. [19] conceptualised MA as ensuring access to healthcare products in order to reduce health inequalities. Rollet et al. [24] and Hughes-Wilson et al. [22] viewed it as patients having access to orphan medicinal products (OMPs). This group of drugs is different from regular drugs and used for treating rare diseases, mostly during clinical trials. OMPs are high-priced and are significantly more expensive than non-orphan drugs. Lordatti et al. [23] evaluated MA as ensured access through the efficacy, safety, and ease of use of drugs. Vialle-Valentin [32] and Waning et al. [30] conceptualized MA in terms of the affordability of the required drug.

Table 2 presents the key findings from the included studies, including the implications of the findings for policymakers, healthcare providers, and pharma. Each study’s findings and its implications were considered in line with how MA was therein conceptualized. Furthermore, how each study ensured access to PP for patients was also considered.

### 3.1. Inductive Analysis of the Included Studies

Identification of the core features or characteristics of MA in pharma is an important step of the analysis that helps to understand how MA is conceptualised and defined. In this study, five conceptual dimensions or themes of MA in pharma were identified from the dataset, namely “right products”, “right patient”, “right price”, “right point” (time), and “right place” (setting). These five distinct themes were identified from the core attributes of MA in pharma across the dataset.

#### 3.1.1. Right Products

From the current review, Larson et al. [20], Rutta et al. [21], Lordatti et al. [23], Rollet et al. [24], Romao et al. [25], Miller et al. [26], and Patouillard et al. [29] conceptualised MA as product availability and accessibility to the patients who need and will benefit from them. Availability in this context means that the required drugs can be used by the patients, as they are there for the patients to use as required, while accessible means that there are no restrictions for the patients to use the available drugs and that the patients can easily reach or obtain the drugs when required. 

Specifically, Lordatti et al. [23] highlighted availability or having the right product as a core attribute of MA. In this hospital-based study conducted in France, the authors considered MA as the physician’s ability to develop his or her own ideas about the value of new drugs based on efficacy and safety. MA is conceptualised as drugs having efficacy, safety, access, and ease of use. Similarly, Romao et al. [25] and Patouillard et al. [29] submitted that MA is about access to pharmaceutical products. Larson et al. [20] also described MA as a process that ensures the uptake of pharmaceutical products or market adoption of new pharmaceutical product. Miller et al. [26] conceptualised MA as granting physicians the right to prescribe even medicinal experimental therapies unapproved by the regulatory authority to terminally ill patients in a bid to provide patients with expanded and quick access to treatments. According to Rutta et al. [21], MA was conceptualised as having access to special drugs such as Artemisinin-based combination therapy (ACT). ACT is generally recommended for the treatment of uncomplicated plasmodium falciparum malaria. Even to a higher degree, Rollet et al. [24] conceptualised MA as the right to treatment including OMPs, which are drugs intended for the diagnosis, prevention, or treatment of life-threatening or very serious conditions. Despite this, OMPs are typically eligible for conditional marketing authorisation. The right products in this review encapsulate drugs of different types and even medical devices, as shown in only one study [25]. Larson et al. [20] viewed MA in the light of product availability, where stocking of new product will promote its prescription and lead to local demand and thus market adoption of new pharmaceutical product. Lordatti et al. [23] submitted that giving physicians information about new PP enabled them to ensure safety, access, efficiency, and drug ease of use, i.e., the route of drug administration. This knowledge helped the physicians to understand contraindications better—how drugs can have side effects when used together—leading to better intervention for patients. Furthermore, Larson et al. [20] stated that empowering isolated shops with the right resources to stock PP could help to ensure better access for patients, recommending enabling health care providers to work with wholesalers so as to ensure access to PP for the patients and urging policymakers to ensure that isolated shops are stocked with PP for patient access. These could have an economic implication for policymakers and health care providers to ensure that the right product goes to the right patients, as revealed in the included studies.

#### 3.1.2. Right Patient

The “right patient” as a theme in this review means that the patients for which a drug was formulated or intended have access to such drugs (i.e., patient with malaria having access to malaria drug or diabetic patients having access to diabetic drugs). Eleven out of the fourteen included studies conceptualised MA as ensuring the right patients have access to PP. Analysis of the studies indicated that MA was about getting the right products to the right patients. Thus, MA as regarding the accessibility of products to the right type of patients was confirmed by Ameh et al. [19], Larson et al. [20], Rutta et al. [21], Hughes-Wilson et al. [22], Iordati et al. [23], Rollet et al. [24], Romao et al. [25], Lee et al. [31], Miller et al. [26], Schmittdiet et al. [27], and Patouillard et al. [29]. 

Ameh et al. [19], in a community-based study conducted in Nigeria, Kenya, and Tanzania, conceptualised MA as unrestricted access for appropriate patients who would benefit from health care products (drugs). Hughes-Wilson et al. [22] conceptualised MA as patients having unrestricted access to orphan drugs in Europe. Lordati et al. [23] conceptualised better education for the physician about the characteristics of the drugs prescribed to patients so as to ensure that the right patients are getting the right drugs in France. Larson et al.’s [20] hospital-based study in Tanzania conceptualised MA as having malaria drugs for the right patients. Rutta et al. [21], in another study in Tanzania, considered access to the use of Artemisinin-based combination therapy (ACT) drugs for patients with malaria. Also, Patouillard et al. [29] conceptualised MA as malaria patients having access to malaria drugs. Lee et al. [31] conceptualised MA as HIV patients having access to paediatric antiretroviral drugs (ARVs) without restrictions in developing countries. Miller et al. [26] considered MA to include expanded access to ensure uptake of PP (drugs) even for terminally ill patients, as it is currently a law approved in 36 states in the United States of America (USA). Rollet et al. [24] conceptualised MA as patients having sustained access to orphan drugs in Europe. Romao et al. [25], in a community-based study in Portugal, described MA as patients having a relationship with pharmacists to access products (or medical devices). Schmittdiet et al.’s [27] study in the USA ensured that diabetic patients had access to the right diabetic drug. This study found that ensuring that the right patients can obtain the right products can help with the societal perspective of health iniquities, and this could also help policymakers and healthcare providers to envision policies that would help to reduce health iniquity and uptake of pharmaceutical products.

#### 3.1.3. Right Point

The “right point” in this review refers to patients having access to PP on time and not when it is too late (i.e., having access at the point). Hughes-Wilson et al. [22] and Rollet et al. [24] conceptualised patients having access to orphan drugs even before such drugs are given authorisation so that the right patients could access the drug during the experimental stage. Patouillard et al. [29], in a study aimed at investigating the determinants of price mark-ups on anti-malarial drugs in retail outlets in Cambodia, measured accessibility as the required travel time to the closest main commercial area with a 4-wheel-drive vehicle. Based on the time needed to travel to have access to anti-malaria drugs, markets were grouped into three categories: “accessible” (markets located less than 2.5 h from the closest commercial area); “moderately accessible” (2.5–4.5 h); and “remote” (more than 4.5 h). In order to ensure rapid and continued access to products, Rutta et al. [21] described Accredited Drug Dispensing Outlet (ADDO) programmes used to ensure access to Artemisinin-based combination therapy (ACT) drugs for patients. Similarly, Lee et al. [31] conceptualised MA in terms of “right point” by ensuring that HIV patients had unrestricted access to antiretroviral drugs as soon as they are diagnosed. Simon et al. [28] highlighted the adoption of computerised order entry (CPOE) in the USA as a platform to ensure MA efficiency, as it is intended to allow patients access to PP at the right time. A total of six of the studies included conceptualised MA as patients having access to PP at the right point. Lee et al. [31] conceptualised patients having better access to PP through technological improvement of the CPOE programme, and this led to better access to PP. Hence, this technological approach can be used by policymakers and health care providers to ensure access to PP. Also, societal perspective changed with the adoption of the CPOE, as participants were hesitant before its introduction, but this perspective changed after its introduction.

#### 3.1.4. Right Price

Ameh et al. [19] conceptualised MA as access to health care products and reducing health inequalities through the four As: availability, accessibility, affordability, and acceptability. In this definition, affordability is about right pricing of the product. Considering MA and pricing, Lee et al. [31] posited that the pricing of drugs should be relative to economic status of countries. The authors found that pharmaceutical price setting for countries with low income helped to ensure uptake of PP. The pricing trends of their analysis explain why low-income countries are paying the lowest originator price, followed by lower-middle-income and upper-middle-income countries. Irrespective of product, pricing seems to be crucial to MA for ensuring that patients will have access to products. In this light, Rollet et al. [24], with respect to access to OMPs, submitted that the cost of manufacturing should determine the fair price of OMPs, as high-priced OMPs exacerbate the affordability problem for health care budgets. Schmittdiel et al. [27] stressed that the cost of drugs can affect access and medication adherence. This was confirmed in their study, where physicians switched patients’ drugs from high-cost drugs to low-cost drugs considering patients’ out-of-pocket costs. Furthermore, Vialle-Valentin [32] described MA in terms of accessibility and affordability. Promoting affordability through the development of national policies to improve health care finance systems was projected to avoid catastrophic health costs. Thus, MA involves ensuring that PP are fairly priced and reimbursed. According to Ameh et al. [19], it is good to consider the cost of PP to ensure access to such products. The included studies that considered the right price helped to reduce the economic burden on patients, thereby increasing uptake of and access to PP. Healthcare providers could adopt this approach to reduce the economic burden of PP uptake for patients.

#### 3.1.5. Right Place

The “right place” in this review refers to the “right setting” and was identified as a key theme and attribute of the MA process that will determine successful patient access to PP. Accordingly, economic, policy, societal, and technological contexts emerge as settings that tend to define MA process. Taking context into consideration, pharmaceutical price setting should vary across countries to ensure uptake of PP. According to Lee et al. (2016), price setting for PP should be country-specific, especially regarding economic status (i.e., low-income nations), to ensure uptake. From their analysis, the pricing trends explain why low-income countries are paying the lowest originator price, followed by lower-middle-income and upper-middle-income countries. Lee et al. [31] posited that manufacturers should consider innovative incentives that would help with the uptake of their products to ensure easy use of their products, which could lead to better access for patients. For example, in low and-middle-income contexts, MA processes for pharmaceutical products involve programmatic approaches aimed at reducing health inequalities.

With respect to policy, some national health services promote the MA process of PP in some settings. For example, in some settings, patients’ access to drugs is based on out-of-pocket spending in other contexts, especially in the low and-middle-income countries. Patouillard et al. [29], in a study conducted in Cambodia, submitted that MA for malaria drugs will require access to PP for free from the government-owned outlets, including health centres and hospitals. Out-of-pocket spending may constitute barriers to patients’ access to potent medicine. For example, in a study conducted in the USA by Schmittdiel et al. [27], physicians switched patients’ drugs from high-cost drugs to low-cost drugs considering patients’ out-of-pocket costs. Furthermore, Romao et al. [25] submitted that the government paying for ostomy products ensured increased access to ostomy products, which ensured access to the right patients. Policymakers and health providers should ensure that they support patients with intestinal ostomy. Also, Rutta et al. [21] suggested for treatment of malaria using combined treatment ACTs, especially in low-income countries where malaria is prevalent, that the government should remove the sale of alternative anti-malarial drugs to increase the uptake of ACT drugs and that this would also ensure safety for the patients. Ameh et al. [19], in a study carried out in Nigeria, Kenya, and Tanzania, conceptualised MA as accessing healthcare products and reducing health inequalities.

Based on the use of technology to improve MA for pharma, Simon et al. [28] recommended use of electronic prescribing as a way of improving the MA process for PP. computerised order entry (CPOE) is obtainable in high-income countries, but other parts of the world still use the handwritten system. According to Simon et al. [28], CPOE was introduced to reduce medication errors from using hand-written prescriptions for patients in an effort to enhance patient safety. It was revealed that it was easier to read a doctor’s prescription via computer than when handwritten. CPOE was reported to be effective when measured in terms of governance, preparation, support, perception, and consequences.

From the societal perspective, Vialle-Valentin et al. [32] submitted that in low-income countries, out-of-pocket expenses on healthcare products are linked to their income. Therefore, community-based health insurance (CHI) initiatives in low-income countries, particularly Rwanda, ensured access to PP for patients. Also, the scheme ensures uninterrupted access to PP if patients were members of the CHI. CHI membership was set by the government at USD 2.5–3.0 per family per month, and it is voluntary, differing between urban and rural areas. Members receive medicine free of charge. The results showed that perception about the quality of care provided by health professionals affected CHI membership. Eighty-five percent of private healthcare expenditures include out-of-pocket payments. Income levels determine the amount of expenditure on medicine. The Ministry of Health is focused on helping very poor patients to get access. The most used medicines are amoxicillin, paracetamol, quinine, cotrimoxazole, and penicillin V6. Another context-specific initiative was reported by Waning et al. [30], where a rural pharmacy initiative (RPI) was established to help more than 300 rural Kyrgyz who were reported to be without physical access to PP in 2004. This was caused by the shortage of pharmacists in the area. Geographical access to a pharmacy was considered a determinant of the health of the community. A non-profit strategy such as RPI helped to expand access to PP. The RPI helped to regulate the price of PP within the pharmacies, leading to cheap PP for both rural and urban patients.

## 4. Discussion

The present scoping review aimed to investigate how is MA is conceptualised and its role defined within pharma. According to Sendyona et al. [12], the concept of MA is still poorly understood, and the definition varies depending on the stakeholders’ perspectives. Twelve out of the fourteen included studies in this review conceptualised MA in pharma as patients having better access to PP, using tools such as pricing, cost, FDA/NICE approvals, efficacy, safety, and medication adherence for ensuring patients access and uptake of PP [19,20,21,22,23,24,25,26,29,30,31,32]. Thus, there is an overwhelming agreement among these studies in defining MA as the patient having the right product at the right time and right price.

Schmittdiel et al. [27] focused on how the cost of drugs can affect access and medication adherence. Similarly, other authors employed terms such as affordability and pricing of medications [19,24,31,32]. Studies agree that pricing is an attribute of the MA process that is important in enabling faster patient access to pharmaceutical products [7,33,34,35]. Thus, it is suggested that pharmaceutical and biotechnology industries develop effective MA strategies that may attract the best possible reimbursement for investments in their product while keeping the products affordable for payers to guarantee patients’ access to new, effective therapies in the fastest possible time [7]. Also, proper regulation of patient medicine vendors (PMV) might help with patients having access to the right pharmaceutical product (PP).

Simon et al. [28] explored how safety and efficacy can ensure access to PP. Larson et al. [20], Rutta et al. [21], Lordatti et al. [23], Rollet et al. [24], Romao et al. [25], Miller et al. [26], and Patouillard et al. [29] conceptualised MA as product availability and accessibility for the patients who need and will benefit from them. Availability in this context means that the required drugs are made available to the patients. All the studies included used various MA strategies to ensure that patients have better access to PP.

Few studies have defined or conceptualised MA in terms of the right patients. Simon et al. [28] classed MA as having the right product for patients. MA has emerged as a crucial element of the pharmaceutical industry [36]. One of the core attributes of MA is getting or making available the right products needed for healthcare to the end users. According to Kumar et al. [37], MA can be used to guarantee that patients have better access to the right pharmaceutical medicines (the right product).

Hughes-Wilson et al. [22] and Rollet et al. [24] conceptualised patients having access to orphan drugs even before such drugs are given authorisation so that the right patients could access the drug during the experimental stage of development to ensure the patient receives the drug at the “right point”. The right point means patients having access to PP on time and not when it is too late for them. Patouillard et al. [29] ensured the right point for the patients in their study was facilitated by making sure PP were made available to patients based on geographical location but irrespective of their location. Rutta et al. [21] made anti-malarial drugs available to patients by training people to diagnose and treat malaria. Lee et al. [31] ensured the right point for people with HIV requiring an anti-retroviral drug, and Simon et al. [28] used the adoption of computerised provider entry (CPOE) to ensure the right point for patients in their study. Being treated in the fastest time and with the appropriate product is an important attribute of MA. Studies agree that MA is the process that ensures that the right product gets to the end user at the right time. It is believed that getting the product to the customer at the right point is one of the challenges to MA in pharma [7,33,34,35]. To stress the importance of time, Khoury [38] suggested that pharmaceutical companies need to reach the right clinician at the right time to impact point-of-care decisions that result in optimal patient outcomes.

In this review, the “right place” was identified as the missing element in the previous attempts at defining MA in pharma, which seems to be conspicuously omitted in the conceptualisation of MA in both the grey and research literature. The right place includes the geographical location and the setting in that geographical location. The conceptualisation of MA in pharma is incomplete without considering the context or setting (i.e., the place). In this review, economic, policy, societal, and technological contexts seem to define the MA process. Across the global north–south divide, MA is conceptualised differently. In Nigeria, Kenya, and Tanzania; Ameh et al. [19] conceptualised MA in pharma in terms of reducing health inequality among patients. Hughes-Wilson et al. [22] ensured access at the right place by making OMP available to patients in Europe. Lee et al. [31] encouraged manufacturers to ensure a global uptake of PP for patients through their MA strategies by making low-income countries pay less for PP. Vialle-Valentina et al. [32] conceived of MA in pharma in low-income countries as involving paying less for PP. Some PP are context-relevant; for example, malaria medicine needs to be accessible to malaria-endemic regions. In Cambodia, Patouillard et al. [29] ensured access to malaria drugs. Also, Rutta et al. [21] ensured better access to malaria drug ACTs in Tanzania. Apart from viewing the right place in a geographical context, it was also considered based on economy. Schmittdied et al. [27] conceptualised access to PP as physicians switching from high-cost drugs to low-cost drugs based on out-of-pocket status to ensure better access to PP in the USA. Romao et al. [25] conceptualised access to PP by funding the cost of ostomy bags so as to ensure access for patients to the product in Portugal. In terms of policy, Waning et al. [30] conceptualised training people, referred to as the rural pharmacy initiative (RPI), to help 300 rural Kyrgyz who were reported to have no access to PP. In general terms, MA refers to the ability of a company to sell goods and services across borders or to enter a foreign market or another country [39]. Thus, MA is not like “free trade” across borders or settings but is subject to the conditions or requirements and negotiations needed for its achievable goal [40,41]. In essence, every setting has its distinctive characteristics.

In sum, 11 out of the 14 studies included in this review considered MA in pharma as ensuring the right patients have access to PP. Analysis of the studies indicated that MA was about getting the right products to the right patients. Thus, MA should concern accessibility of the right product for the patient [19,20,21,22,23,24,25,26,27,29,31]. Other MA strategies include the cost of PP [28], medication adherence (Schmittdiel et al., 2010), community-based health insurance (CHI) [32], affordability and rural pharmacy initiative (RPI) [30], CPOE [28], ADDO [20,21], OMPs [24], trained volunteers referred to as village malaria workers [29], “expanded access” and “compassionate use” programmes for PP [26], pricing trends within the paediatric ARV market [31], and the four As of access, namely available, accessible, affordable, and acceptable [19]. The aforementioned are in line with the conclusion of PRMA consulting [7] that MA is about the right patients getting the right PP at the right time and, if possible, at the right price.

Market access can be used to ensure that the appropriate patients have better access to the right pharmaceutical medicines at the right time and at the right price [37]. In order to produce evidence relating to “patients’ demands, safety, efficacy, effectiveness, budget impact, and cost-efficiency of the technology as compared with existing treatment alternatives”, MA has emerged as a crucial element of the pharmaceutical industry [36]. According to the health technology assessment (HTA), manufacturers must overcome a number of external obstacles to ensure successful product commercialisation due to budgetary restraints and an increased reliance on formal HTA [36]. Due to these requirements, pharmaceutical corporations have been forced to coordinate the production of the necessary proof internally [36]. As a result, pharmaceutical companies now employ MA professionals [37,42]. In view of this, Sendyona et al. [12] (p. 1) claimed that “a broader grasp of MA and the value perspectives of the many stakeholders is necessary”. Following this scoping review, we have a better understanding of how MA is conceptualised and offer a new definition:

*MA in pharma concerns providing the right products for the right patients, delivered at the right point in time, within the right place or setting, and at the right price.*

### Strengths and Limitations

The studies included in this review were not assessed in terms of methodological quality, as this is not a requirement for scoping reviews due to the broad range of literature included [14]. Nonetheless, this could be seen as a limitation. Another limitation of this study was that only studies carried out in the English language were included, and it is possible to have omitted relevant studies conducted in other languages. However, this is the first review on how MA is conceptualised and its role defined within pharma beyond grey literature reports. Hence, this study serves as a reference source in the emerging field of MA in pharma as well as contributing to its epistemology.

## 5. Conclusions

Market access in pharma can be summarised as a process that commences with the development and availability of the right products that are proven to be efficacious and disease/condition-specific; are specifically produced for the right patients or end users who will maximise the best clinical outcomes and economic value; are delivered at the right point in a timely, sustained, and efficient manner; are given at the right price (commercially viable or reimbursed price that represents good value); and are conducted within the economic, policy, societal, and technological contexts, with the overarching goal of achieving the best patient outcomes and ensuring product profitability. Further research is needed to better understand the MA professional’s role in cognisance of this new understanding.

## Figures and Tables

**Table 1:** Characteristics of included studies.

Authors, Year	Study’s Methodology	How Is MA Conceptualised?	How Is MA Defined?	Type of Pharmaceutical Product	MA Strategy	Setting	Country of Study	Disease Areas
**Africa Region**								
Ameh et al. [19]	Study design—Community-based qualitative studies Sample size—FGD (N = 155), IDI (N = 25), and KII (N = 11)	Access to health care products and reducing health inequalities	Ensuring access for patients through the 4 As—availability, accessibility, affordability, and acceptability	Drug	Access	Community	Nigeria, Kenya, and Tanzania	N/A
Larson et al. [20]	Study design—Longitudinal survey of ADDO Sample size—356 ADDOs	Prescription of medicine relating to uptake of pharmaceutical products and hence market adoption of new pharmaceutical product	Stocking of new products will lead to local demand for effective products	Drug	Access	Community	Tanzania	Malaria
Rutta et al. [21]	Study design—Retrospective analysis of ADDO model Sample size—448 ADDO dispensers	Access to Artemisinin-based combination therapy (ACT) drugs for patients	Accredited Drug Dispensing Outlet (ADDO) programs were used to ensure access for patients	Drug	Access	Community	Tanzania	Malaria
Vialle-Valentin et al. [32]	Study design—Secondary data analysis Sample size—1267 individuals	Access and affordability	Development of national policies to improve health care finance system to avoid catastrophic health cost	Drug	Access	NA	Low-income countries, with Rwandan experience	N/A
**America Region**								
Schmittdiel et al. [27]	Study design—Cross-sectional Survey Sample size—1458 Medicare beneficiaries with diabetes	How the cost of drugs can affect access and medication adherence	Physicians switched patients’ drugs from high-cost drugs to low-cost drugs considering patients’ out-of-pocket costs	Drug	Cost and adherence of drugs	Community	USA	Diabetes
Simon et al. [28]	Study design—Qualitative study Sample size—IDI (N = 24)	Computerised order entry (CPOE) to ensure safety, quality, and efficiency for patients.	Hospitals in USA rapidly adopted CPOE to reduce errors with medicines and to ensure safety and efficacy	Drug	Safety and efficacy	Hospital	USA	N/A
**Asia Region**								
Patouillard et al. [29]	Study design—Qualitative study Sample size—38 sub-districts Each sub-district provides PHC services to around 10,000 inhabitants.	Access to pharmaceutical products free from the government-owned outlets, including health centres and hospitals	Using economic perspective, 3 categories of MA was established to determine product price: (1) Accessible market—less than 2.5 h, (2) moderately accessible market—2.5—4.5 h, and (3) remote market—more than 4.5 h	Drug	Access	Community	Cambodia	Malaria
Waning et al. [30]	Study design—Secondary data analysis Sample size—N = 162,999 data	Affordability of medicine	The Kyrgyz ministry of health changed the law in 2005 to let nurses fill prescriptions with chemists in rural areas after completing 2-week training program	Drug	Access	Hospital	Kyrgyzstan—Central Asia	N/A
**Europe Region**								
Lordatti et al. [23]	Study design—Mixed method Sample size—NA	Efficacy, safety, access, and ease of use	Physicians to develop their own ideas about the value of new drugs based on efficacy and safety	Drug	Access	Hospital	France	N/A
Hughes-Wilson W. et al. [22]	Study design—Secondary data analysis Sample size—NA	Access to orphan drugs for patients	These should include rarity, disease severity, the availability of other alternatives (level of unmet medical need), and the level of impact on the condition that the new treatment offers	Drug	Access	Global	Europe	N/A
Rollet et al. [24]	Study design—Secondary data analysis Sample size—NA	Access to orphan medicinal products (OMPs)	Cost of manufacturing should determine the fair price of OMPs. High-priced OMPs exacerbate affordability problem for health care budget	Drug	Access	Global	Italy, Spain, and Germany	Oncology—Cancer
Romao et al. [25]	Study design—observational, cross-sectional, multicentre study Sample size—185 pharmacies; 412 people	Access to pharmaceutical products and patients having relationship with pharmacist	In 2017, the government enacted a law to improve the control of and equitable access to pharmaceutical products	Medical device	Access	Community	Portugal	Ostomy patients
**Unspecified**								
Lee et al. [31]	Study design—Narrative review Sample size—NA	Pharmaceutical price setting for countries with low income to ensure uptake of pharmaceutical products	Designed to know how prices of drugs are determined. The pricing trends of their analysis explain why low-income countries are paying the lowest originator price, followed by lower-middle-income and upper-middle-income countries.	Drug	Access	Global	N/A	HIV
Miller et al. [26]	Study design—Retrospective data analysis Sample size—398 programmes	Expand access to ensure uptake of pharmaceutical products	Allow physicians to prescribe medicinal experimental therapy unapproved by the USA Food and Drug Administration (FDA) to terminally ill patients—this law is now approved in 36 states so as to ensure access.	Drug	Access	Hospital	N/A	HIV, leukaemia, and multiple myeloma

Key: ADDO, Accredited Drug Dispensing Outlets; FGD, focus group discussion; IDI, in-depth interview; KII, key informant interview; LMIC, low- and middle-income countries; NA, not available/not applicable; PHC, primary health care; USA—United States of America.

**Table 2:** Key findings and implications of the reviewed studies.

Authors’, Year	Key Findings	Implications	How MA Is Conceptualised
Ameh et al. [19]	Patients having free health services and insurance had better access to pharmaceutical products (PP). However, patient medicine vendors (PMV) were perceived to be more affordable/accessible than those provided by health providers. PMV were also flexible with instalment payment for service fee.	The cost of pharmaceutical products is an important consideration in ensuring access to PP. Also, proper regulation of PMV improves the patient’s access to the PP. Hence, there is a need for policy and programmatic actions to regulate PMV to ensure safety and access to PP for patients.	MA is conceptualized in terms of affordability (right price) and availability of PP at the right context or place using free health services, insurance schemes, and PMVs.
Hughes-Wilson W. et al. [22]	Patients’ access to orphan drugs and availability of other alternative medication and new treatments for rare and severe diseases to meet unmet medical needs.	Increasing budget on orphan drugs will improve availability and affordability of product for the indicated patients for which the orphan drugs are produced.	MA is conceptualised in terms of right product (orphan drugs) at an affordable cost (right price) for the right patients (rare disease).
Lordatti et al. [23]	Giving physicians information about new PP improves knowledge of benefits, contra-indication, and use of the product to ensure safety, access, and efficiency of the PP.	Training of health care professionals such as physicians on the use of new PP in order to improve better patient access as well as ensure safety and efficacy for the patients using the new drug introduced.	MA is conceptualized in terms of the availability (right product) of a new product at the right point (following MAu) through the right setting/medium (physicians).
Larson et al. [20]	Stocking wholesalers with higher numbers of proximal shops and clinics, larger customer traffic, and the presence of a licensed pharmacist in densely populated areas with subsidized anti-malarial drugs (ACT) rather than having isolated shops that serve fewer customers played a major role in expanding PP availability.	Empowering wholesalers with higher market competition and customer demand metrics with the right and subsidized resources to stock PP ensures better access for patients in developing country markets. Healthcare providers work with wholesalers to ensure access to PP for the patients.	MA is conceptualized in terms of the availability (right product) and affordability of PP (right price) supplied through high-profile shops in competitive markets and wholesale suppliers to ensure faster product diffusion across all drug retailers (right place).
Lee et al. [31]	Sub-Saharan Africa (SSA) (excluding South Africa) pays the lowest price for antiretroviral drugs. East Asia and the Pacific and South Asia were pays 10% less than SSA. Lower-, middle-, and upper-middle-income countries pay higher costs for PP. Also, availability of child-friendly type of formulation of drugs played a major role in the uptake of PP.	Pharmaceutical industries’ innovative incentives based on geographical considerations as well as product available based on age demographics improve uptake of products and better patient access.	MA is conceptualized in terms of the availability (right product) and affordability of PP (right price) based on geographical considerations (right place).
Miller et al. [26]	Benefits, limitations, and ethical and regulatory implications of programmes involving expanded access and compassionate use of experimental therapies to terminally ill patients as well as the proportion of such programmes that ultimately received MAu were highlighted.	Allowing healthcare providers to use safe and efficacious drugs not yet approved by regulatory bodies like FDA in America might promote better access to PP by patients.	MA is conceptualized in terms of the availability (right product) of PP using expanded and compassionate access programmes for terminally ill patients (right patient).
Patouillard et al. [29]	Price mark-ups, which are influenced by several contextual factors as well as other key elements of anti-malarial supply and demand, play an important role in the limited access to appropriate anti-malarial drugs in retail outlets in Cambodia.	Reducing the cost of medications and regulating price mark-ups by printing the price of the product on the pack may translate to better patient access and reduce out-of-pocket expenses.	MA is conceptualized in terms of availability (right product) and affordability (right price) as well as consideration for the economic context/setting (right place).
Rollet et al [24]	Publicly available national statistics showed that the budget impact of orphan medicinal products (OMPs) is low due to small population size and might plateau in 5 years’ time. OMPs are mostly for oncology treatment.	Increasing the budget on OMPs will help with access and affordability for the patients to the orphan drugs that are produced.	MA is conceptualized in terms of availability (right product) and affordability (right price) of orphan medicinal products for the right patient.
Romao et al. [25]	Portuguese National Health Service (NHS) provided coverage for ostomy product, without out-of-pocket payment, thus providing better access to this medical device (ostomy products).	Government paying for ostomy products ensured increased access to ostomy products to support patients with intestinal ostomy.	MA is conceptualized in terms of availability (right product) and affordability (right price).
Rutta et al. [21]	Transition from monotherapies to the use of the combined treatment artemisinin-based combination therapy (ACT) promotes better access, safety, and efficacy of PP. The transition also helped to give access to underserved populations. Also, sales of alternative anti-malarial drugs were impacted.	A better product with proven efficacy and regulation or ban of alternative or less efficacious products increase uptake of the product. Thus, the availability of combined treatment ACTs, especially in low-income countries where malaria is prevalent, improves patient access.	MA is conceptualized in terms of the availability (right product) of PP.
Schmittdiel et al. [27]	Medicare Part D beneficiaries with diabetes who entered the coverage gap have low levels of communication with physicians about drug costs despite the high perceived importance of such communication.	Patients with chronic conditions taking multiple drugs who are at risk of high costs of drug may benefit from physician–patients communication about the cost of medicines and cheaper alternatives so as to ensure access to PP.	MA is conceptualized in terms of availability (right product) and affordability (right price) of PP, as the cost of products can affect access and medication adherence.
Simon et al. [28]	Computerised order entry (CPOE) was introduced to reduce medication errors associated with handwritten prescriptions as well as increase safety for patients. CPOE was effective due to the following: governance, preparation, support, perception, and consequences.	COPE represents a meaningful use of health information technology for prescribing, as healthcare professionals enter accurate and complete medication orders electronically and so reduce medication errors and subsequent adverse drug reactions.	MA is conceptualized in terms of the availability (right product) of PP for the right person.
Vialle-Valentin et al. [32]	Community-based health insurance (CHI) schemes expand access to medicines in low-income countries.	CHI has the potential to improve access to, affordability of, and use of medicines at the household level in low-income countries.	MA is conceptualized in terms of the availability (right product) and affordability of PP (right price).
Waning et al. [30]	The rural pharmacy initiative (RPI) increases equitable access to products in rural regions by acting as a market driver, stimulating competition in medicine prices in competitor pharmacies, even when they were located in different villages.	An RPI scheme increases access to medicines in rural settings by impacting price competition.	Affordability of medicine.
